# Draft Genome Sequences of Vancomycin-Intermediate *Staphylococcus aureus* Strains in South Korea

**DOI:** 10.1128/genomeA.00027-16

**Published:** 2016-06-16

**Authors:** Jung Wook Kim, Jae Il Yoo, Gi Su Kang, Yeong Seon Lee, Jae-Yon Yu, Chan Park, Il-Hwan Kim

**Affiliations:** Division of Antimicrobial Resistance, Center for Infectious Diseases, National Research Institute of Health, Korea Centers for Disease Control and Prevention, Cheongju, South Korea

## Abstract

We report here the draft genome sequences of four vancomycin-intermediate *Staphylococcus aureus* (VISA) strains from South Korean hospitals participating in a nationwide laboratory surveillance program for vancomycin-intermediate and vancomycin-resistant *Staphylococcus aureus*. All strains harbor mutations in the *walKR*, *graSR*, and/or *rpoB* genes that are known frequently mutated determinants of VISA.

## GENOME ANNOUNCEMENT

The use of vancomycin for the treatment of methicillin-resistant *Staphylococcus aureus* (MRSA) infections (1) has been challenged by the emergence of vancomycin-intermediate and vancomycin-resistant (2, 3) *S. aureus* (VISA and VRSA, respectively). We report here on four VISA strains (V040, V808, V1127, and V1142) from South Korean hospitals participating in a nationwide laboratory surveillance program for VISA/VRSA. To provide insight into the VISA mechanism, we selected representative strains from each multilocus sequence type (ST), staphylococcal cassette chromosome *mec* element (SCC*mec*), and accessory gene regulator (*agr*) type for whole-genome sequencing. Strains V040, V808, V1127, and V1142 belong to ST72-SCC*mec* IV-*agr* I, ST239-SCC*mec* III-*agr* I, ST5-SCC*mec* II-*agr* II, and ST1-SCC*mec* IV-*agr* III, respectively. All strains have a vancomycin MIC of 4 µg/ml, and V040 additionally has a teicoplanin-resistant (MIC, 24 µg/ml) phenotype.

Whole-genome sequencing for all strains was performed using an Illumina MiSeq or Illumina HiSeq platform. The sequencing library was prepared with the TruSeq DNA LT sample prep kit (Illumina, CA), according to the manufacturer’s instructions. The generated paired-end sequencing reads were assembled using CLC Genomics Workbench 7.3 (CLC bio, Denmark). Prediction of genes was performed using Glimmer 3 (4), and annotation was conducted by homology search against the Clusters of Orthologous Groups (COG) and SEED databases (5, 6). As validation, the assembled sequences were compared with the MRSA reference genomes of N315 and Mu50. The draft genome information for all strains is summarized in Table 1.

**TABLE 1:** Draft genome information

Strain	Coverage (×)	No. of contigs	Genome length (bp)	G+C content (%)	Accession no.
V040	632.57	33	2,727,097	32.73	LFEC00000000
V808	757.79	79	2,769,383	32.63	LFED00000000
V1127	544.59	49	2,863,381	32.84	LFEA00000000
V1142	1,105.44	90	2,602,834	32.69	LFEB00000000

The most frequently mutated determinants in clinical VISA are the *walKR* (7), *vraSR* (8, 9), *graSR* (10, 11), and *rpoB* (12, 13) genes. The V808 strain harbors *walK* (R222K and A468T), *rpo*C (V404I), and *graR* (D148Q) mutations. V1142 harbors *vraT* (E156G), *graR* (F151L), and *rpoB* (T553I) mutations. All strains except V1127 harbor a *graS* mutation (T224I). V1127 harbors an *rpoB* (H481Y) mutation, which is associated with rifampin resistance (MIC, >32 µg/ml).

### Nucleotide sequence accession numbers.

These whole-genome sequences of *S. aureus* have been deposited in NCBI GenBank under the accession numbers described in Table 1.
